# Supplementary material for: Identification of two genomic cryptotypes of Plasmodium malariae in Africa
Source: PLoS Negl Trop Dis. 2026 Jul 15;20(7):e0014176. doi: 10.1371/journal.pntd.0014176 (PMC13387610; doi:10.1371/journal.pntd.0014176)
Supplement: Attachment — Submitted filename: Answer_reviews.pdf [file pntd.0014176.s013.pdf]

Dr. Margaux Lefebvre
MiVEGEC Research Unit (Université de Montpellier, CNRS, IRD)
911 Avenue Agropolis, 34394 Montpellier Cedex 5, France
Max Planck Institute for Evolutionary Anthropology
Deutscher Platz 6, 04103 Leipzig, Deutschland
margaux.jm.lefebvre@gmail.com, margaux_lefebvre@eva.mpg.de

8th of June 2026

Dear Editor,

Please find enclosed a revised version of our manuscript (PNTD-D-26-00567) entitled "Identification of two genomic cryptotypes of *Plasmodium malariae* in Africa" by Lefebvre and colleagues.

We thank the reviewers for their constructive and helpful comments on the manuscript, which significantly helped us improve its quality and the strength of its conclusions. We have modified our manuscript accordingly, following the reviewers comments as much as possible. The details and point-by-point answers to the reviewers are provided in the attached file.

We hope you will find this manuscript greatly improved and suitable for publication in *PLOS Neglected Tropical Diseases*.

Yours sincerely,

Margaux J.M. Lefebvre
(on behalf of all authors)

We thank the reviewers for the quality of their reviews as well as the high level of expertise, which significantly helped us improve the quality of our manuscript.
Hereafter are our point-by-point responses to the reviewers' comments. We use specific typography to highlight the reviewers' comments (**black**), our response (**blue**), and our corrections in the manuscript (in blue).

Reviewers' Comments and answers :

Reviewer #1: The joining of the NHP and human data doesn't make clear sense. There is a very solid analysis of P. malariae population structure in humans in Africa and a novel observation of P. brasilianum in NHPs in South America. Aside from the observation that there is P. brasilianum in NHPs there seems to be no real value from including the P. brasilianum data here. The major finding (and title) of the paper is not reliant on this data and it is confusing to include here. I would suggest the authors consider whether the article would be better served by separating these two datasets and only focusing on the malariae data here.

R1. We thank the reviewer for this comment. The inclusion of the South American non-human primate (NHP) dataset was not intended as an independent population genomic study of *Plasmodium brasilianum*, but rather as part of a broader effort to characterize diversity across the *P. malariae–P. brasilianum* complex. At the start of the project, one of our objectives was to increase genomic representation of both African *P. malariae* and South American *P. malariae* and *P. brasilianum* in order to investigate their evolutionary relationships and potential host-associated diversification. To achieve this, we screened 226 NHP samples from Colombia and French Guiana and identified 20 *P. brasilianum* infections. Unfortunately, despite successful parasite detection, only two samples yielded genome data of sufficient quality for inclusion in population genomic analyses, reflecting the well-known technical challenges associated with sequencing low-parasitemia infections from wildlife hosts. Although the limited number of high-quality *P. brasilianum* genomes precluded detailed population genomic analyses, these samples still provide important context for interpreting the diversity of the *P. malariae–P. brasilianum* complex. In particular, they reveal that *P. brasilianum* is genetically closer to one of the two African *P. malariae* cryptotypes, whereas American *P. malariae* isolates are more closely related to the other. This observation contributes to the discussion of the evolutionary history and diversification of this parasite complex and raises new hypotheses regarding historical dispersal and host-associated transmission cycles. To address the reviewer's concern, we have clarified throughout the Introduction, Results and Discussion that the primary focus of the manuscript is the population genomic structure of African *P. malariae,* while the *P. brasilianum* component provides complementary evolutionary context rather than constituting a separate population genomic analysis (lines 132-136, 382-385 and 578-582). We also note that Reviewer 2 highlighted this aspect as a particular strength of the study. Therefore, we prefer to keep these data in the manuscript.

Reviewer #1: can the authors clarify what they mean by cryptotype in this context? The P. falciparum observation was a small number of loci around the genome where LD was maintained despite being on different chromosomes. In P. ovale the observation is that there are two "sibling" species which are sympatric but rarely recombine. The identification of

segregation across the first few PCs seems closer to the ovale scenario, though latter data less so. A very clear definition of what is expected for each scenario would be valuable.

R2. We thank the reviewer for this comment. In the present study, we use the term cryptotype to refer to a previously unrecognized genetic lineage within African *P. malariae*. While *P. malariae* in Africa was previously considered to consist of a single genetic cluster, our analyses identify two genetically distinct and sympatric sub-clusters that nevertheless do recombine with one another. Unlike the situation described for *P. ovalecurtisi* and *P. ovalewallikeri*, which represent distinct sympatric species with little or no evidence of recombination, the two African *P. malariae* clusters identified here show clear evidence of admixture and ongoing recombination. We therefore do not interpret them as separate species or subspecies. Our usage is closer to the concept proposed by Miotto et al. (2024) for African *P. falciparum*, where cryptotypes correspond to distinct genetic backgrounds that persist within a recombining population despite extensive geographic overlap. However, the *P. malariae* clusters described here exhibit stronger genome-wide differentiation than the AF1 cryptotype reported in *P. falciparum*, as they are clearly visible in PCA analyses and ancestry inference. We have added this definition to the manuscript (lines 608-628).

Reviewer #1: it is stated that no biological or geographical criteria allow these clusters to be distinguished. What factors were assessed, in addition what technical factors were assessed (i.e. sequencing depth, sequencing batch, instrument, etc). Do the clusters related to transmission intensity, mosquito species prevalence or historical use of antimalarials?

R3. We thank the reviewer for this comment. This is indeed true that both technical and biological explanations should be carefully considered when interpreting the observed population structure.

First, we did assess whether the observed substructure could be explained by technical artefacts. We found no evidence supporting this hypothesis. Importantly, each cluster (R, Y, and admixed samples) includes isolates originating from multiple independent studies, and therefore from different sequencing batches. In addition, average sequencing coverage varied substantially within each group, with both low- and high-coverage genomes represented in all clusters (cluster R: 1 – 125.3 X; cluster Y: 1 – 732 X; admixed: 1.7 – 409.2 X). Likewise, the clusters contained samples generated using both newly produced and previously published datasets. We have clarified this point in the manuscript (lines 465-469, 601-604, and S2 Table).

Regarding biological explanations, we acknowledge that the current metadata do not allow us to formally test most hypotheses. The majority of isolates originated from infected travelers, and information is generally limited to the country of infection. Consequently, we lack precise data regarding local transmission intensity, mosquito vector composition, environmental conditions, and antimalarial intervention history at the infection site. In the Discussion, we primarily highlight ecological heterogeneity as a possible explanation because the relative frequencies of the two clusters appear to differ across broad ecological regions of Africa. However, ecological variation determines other factors, including mosquito vector species presence/density, transmission intensity, and pathogen prevalence. These factors therefore also represent non-mutually exclusive hypotheses for further investigation. We have therefore revised the manuscript to clarify this point (lines 641-658).

Finally, we agree that antimalarial drug pressure represents an interesting hypothesis. In support of this possibility, one of the strongest signals of differentiation identified in our analyses involves the *DHFR-TS* locus, a gene previously associated with antifolate resistance in *Plasmodium*. Therefore, the impact of antimalarial treatment on the differentiation between clusters is also an interesting hypothesis, which we have now incorporated into the manuscript (lines 699-708).

Reviewer #1:
Figure 4: Was K=2 the best performing value of K?
Line 444-453: Did you examine if the selection of samples results in a geographical separation. There is higher yellow ancestry in East Africa than West Africa. Could the samples used for this analysis be marked on Fig. 3?
You state in the discussion that haplotype based signatures of selection are powerful for detecting recent events – why were they not used here?
Line 129: densify should be replaced with an alternative word.
Figure 3: If the best K is 6, why are the pie charts shown from K=3?
Figure 4: Using shades of red and yellow to define countries and red and yellow to define groups which are not related to geography is confusing. Could the country colors come from a different palate?

R4. We are grateful to the reviewer for these minor comments, which have enabled us to improve the quality of the text and figures in the manuscript.
For Figure 4, K = 2 was identified as the optimal value according to the method of Meisner and Albrechtsen [44]. We have revised the manuscript to clarify this and added a figure illustrating how the variance changes with increasing values of K (S5 Fig.).
We explored the possibility of geographic structuring by testing the relationship between ancestry coefficients and longitude. Although significant associations were detected (R = -0.26 for cluster Y and R = 0.27 for cluster R; *p-value* < 0.001), longitude explained less than 7% of the variance in ancestry coefficients. Visual inspection of the data revealed substantial overlap between the two clusters across Africa, with both clusters represented throughout most of the sampled longitudinal range. We therefore interpret this pattern as a weak east-west gradient in cluster frequencies rather than evidence of strong geographic segregation. We added this result in the manuscript (see lines 471-477 and S6 Fig.).
We have chosen not to mark the samples in Figure 3, as this would risk overloading an already information-dense figure. Instead, we have added an S2 Table listing the African samples and their corresponding cluster assignments. We also have modified Figure 3 to show the pie charts for K=6.
We have also incorporated results from haplotype-based selection scans (lines 355-362, 500-509, and S9 Fig.).
In response to Reviewer 2's suggestions, we have simplified the colour scheme in Figure 2, allowing the African clusters to be distinguished using different shades. However, we have not introduced additional colours, as the existing palette is already assigned to other global regions (Fig. 1 and 2), and further changes could reduce overall clarity.

Reviewer #2: It is becoming increasingly clear that infections by P. malariae are by no means rare (at least in Africa). It is also known that these parasites cause infections that can persist for years. Thus, it is becoming important to study this species in the context of efforts to eliminate malaria. Yet, this parasite species has been relatively poorly studied, and molecular investigations focusing on this parasite are few.
The authors present a very interesting set of analyses of P. malariae populations, based on whole genome sequences, that substantially extends and improves on a recent analysis by Ibrahim et al. (2024). In addition to previous sequenced genomes, those of 45 new isolates from Africa were added, and of particular novelty the authors provide the genomes of two additional P. brasilianum isolates one from Colombia and another from French Guiana.
Their analyses confirmed previous observations concerning the differences between the populations of P. malariae across the continents and interestingly revealed the occurrence of two sympatric genetically distinct groups within the African isolates, which the authors consider as cryptotypes that nonetheless recombine.
The manuscript is well written, the data is clearly and convincingly presented, and fully supports the conclusions drawn by the authors. The salient critique I have is that the discussion presented in the manuscript deserves to be expanded to confront the data with some of the parasitological parameters of P. malariae. As it stands, the discussion generally reiterates the results presented, with little substantive speculation as to the nature or origin of the two cryptotypes.

R5. We thank the reviewer for this summary. We have followed the reviewer's recommendations and expanded on the discussion (lines 615-636, 641-658, 674-688 and 699-715). In particular, we now discuss several non-mutually exclusive hypotheses that may contribute to the maintenance of these cryptotypes, including ecological heterogeneity across Africa, differences in mosquito vector communities and transmission dynamics, adaptation to distinct vertebrate host populations, and variation in malaria control interventions and antimalarial drug pressure. We also discuss the potential relevance of the cluster-specific signatures of selection identified in genes involved in host–parasite and vector–parasite interactions. While the available metadata do not allow these hypotheses to be formally tested, we believe that these additions provide a broader biological and evolutionary framework for interpreting the observed population structure.

Reviewer #2: Why were the two P. malariae-like parasites (Rutledge et al. 2017), as well as the P. brasilianum genome that was previously obtained from a Bolivian strain (Bajic et al. 2022), not included with the other 179 isolates in the main analysis? These omissions are regrettable and somewhat weaken the manuscript, and I suggest that they should be added. This is because it would have helped resolve the phylogeny of the P. malariae-like parasite from chimpanzees and to provide a comparison of the 3 P. brasilianum isolates that were collected in distant geographical regions.

R6. We thank the reviewer for this comment. The two *P. malariae*-like parasites reported by Rutledge *et al.* (2017) were indeed included in our dataset (Table S1) and used as outgroups in the maximum likelihood tree presented in Figure 2B. However, they were excluded from the remaining analyses because they are highly genetically divergent from *P. malariae* and only two samples are currently available. Consequently, they are informative for rooting the phylogeny but do not provide sufficient statistical power for population genomic analyses. Regarding the *P. brasilianum* genome published by Bajic *et al.* (2022), we agree that

additional *P. brasilianum* genomes would be highly valuable. However, this sample is currently available only as a reference genome assembly and not as raw sequencing reads. Most of our analyses relied on genotype likelihood-based approaches that explicitly incorporate sequencing depth and base-quality information through Phred quality scores. Including a sample available solely in FASTA format, and therefore lacking these quality metrics, could introduce methodological bias into the dataset. We nevertheless fully agree that future studies incorporating additional well-characterized *P. brasilianum* genomes from different geographic regions will be important for clarifying the evolutionary relationships within the *P. malariae–P. brasilianum* complex.

Reviewer #2: The speculations concerning the cause of divergence between the two cryptotypes are based on the identifications of 315 genes that appear to have been differentially selected in the two populations (112 Y and 203 for R). For the discussion the authors selected only 22 of these genes, as they are known to be, or extrapolated as, implicated in host-parasite interactions. Speculation as to the nature of the selective forces were limited to a few lines (587-596). This is a major weakness of this manuscript, since this differential selection is what is the most interesting aspect of the manuscript. It is clear that the authors cannot speculate on the vast majority of the genes they identified since their function is unknown or only ascribed putatively. However, there are many avenues that would have been important to discuss.
First, there are numerous antigens that are considered to be important in the acquisition/evasion of immunity with some displaying significant diversity. It is of interest that none were identified in the analysis presented in S2 Table. It would have been of distinct interest to compare the diversity of such genes between the isolates from the two clusters (presumably these could be easily identified from the whole genome sequences).

R7. We thank the reviewer for this valuable suggestion**.** We agree that a comparative analysis of well-characterised antigen genes involved in immune acquisition and evasion would be of considerable interest. However, such analyses would require a dedicated investigation extending beyond the scope of the present study, which was designed primarily to characterize population structure and identify genome-wide signatures of differentiation and selection. Although we did not perform a systematic comparison of diversity across all known antigen genes, our selection scans nevertheless identified signals involving genes implicated in host-parasite interactions. In particular, the incorporation of haplotype-based approaches (*XP-EHH* and *Rsb*) that rely on differences in cluster specific homozygosity (therefore genetic diversity) revealed evidence of selection at the AMA1 locus, a well-characterized antigen involved in erythrocyte invasion and immune recognition. We have now highlighted this result in the manuscript and expanded the discussion accordingly (lines 528-534 and 672-688). We thus agree that a comprehensive analysis of antigen diversity between the two cryptotypes would be highly informative and may help determine whether differences in immune-mediated selection contribute to the maintenance of these lineages. We have therefore included this point as a perspective for future investigations.

Reviewer #2: Second, what the evidence that the two cryptotypes recombine. Is it possible that recombination is rare? Are mixed infections (Y+R) common? If the two types recombine frequently, one would expect them to merge into a single type eventually. If they do not, might this be a vase of speciation? This deserves speculation.

R8. We thank the reviewer for this comment. Several African samples display intermediate admixed ancestry profiles between the two African clusters (~40–60% ancestry from each cluster) while simultaneously exhibiting high F_{WS} values ($F_{WS} > 0.85$), indicating that they represent single parasite genotypes. The most parsimonious explanation is that recombination occurs between the two groups. We have clarified this point in the manuscript (lines 621-628). While these admixed genomes demonstrate that recombination occurs, our data do not allow us to estimate its frequency and we therefore cannot exclude the possibility that recombination remains relatively infrequent. We added this speculation in the manuscript (lines 621-628). Regarding co-infections, samples identified as multiclonal infections were excluded during filtering. Consequently, we were unable to assess whether co-infections involving parasites from both clusters were present.

Reviewer #2: Third, there are some data on the transmission of P. malariae (and P. brasilianum) by different mosquito species. Could the authors speculate as to the differences in the anopheline species that occur between the different regions of the African continent, and whether this could have contributed to the generation of two cryptotypes (admixture of the two parasite types could have been recent, as large population movement between West and East Africa is likely to be recent).

R9. We agree with the reviewer and have added a discussion of this point to the manuscript (lines 641-665). In particular, we note that African malaria transmission is sustained by a diverse assemblage of *Anopheles* species whose geographic distributions vary substantially across the continent. Differences in vector composition, abundance, and ecology could generate distinct selective pressures on parasite genes involved in mosquito-stage development and transmission. This hypothesis is particularly relevant given that several of the genes identified as being under selection in our study are implicated in vector-parasite interactions. Although the available metadata do not allow us to formally test associations between cryptotypes and mosquito species distributions, future studies integrating parasite genomic data with entomological surveys may help determine whether vector-mediated adaptation contributes to the maintenance of these two genetic backgrounds.

Reviewer #2: Fourth, the genetic make-up of the human hosts might have been another contributory factor. Of note the distribution of haemoglobinopathies differ across the African continent. This is also the case for Duffy negativity. Indeed, there was recent speculation (Culleton et al. 2023 Trends in Parasitology, in which one of the co-authors of this manuscript is also a co-author) that P. malariae might preferentially invade reticulocytes. Although infection of Duffy negative individuals by P. malariae occurs, the course of the infection might differ between Duffy negative and Duffy positive persons. It would be of interest for the authors to indicate the ethnic origin of the patients from whom the samples were collected. Since nearly all the African samples collected were from persons returning to the UK or France, it is possible that some could have been of African descent while others not (I am aware that this might be difficult for the samples collected in the UK).

R10. We thank the reviewer for raising this point. Unfortunately, we do not have access to this information regarding the ethnic background, ancestry, or host genetic characteristics of the infected individuals. The samples analyzed in this study were obtained through malaria surveillance programs and were fully anonymized prior to genomic analysis. Consequently, the only metadata available to us is the country visited by the patient. Nevertheless, we

agree that host genetic variation represents a plausible factor contributing to the maintenance of the two cryptotypes. Human populations across Africa differ in the frequency of several malaria-relevant traits, including haemoglobinopathies and Duffy negativity. Such variation could generate heterogeneous selective environments for parasite populations and potentially contribute to the differentiation observed between the two African cryptotypes. This possibility is particularly intriguing in light of recent hypotheses suggesting that *P. malariae* may exhibit specific erythrocyte invasion preferences. Although our dataset does not allow these hypotheses to be formally evaluated, we have expanded the Discussion to acknowledge host genetic variation as a potential contributor to the observed population structure and as an important avenue for future investigation (lines 675-688).

Reviewer #2: Minor comments

Line 89 It is now recognised that the population of P. ovale is actually composed of two distinct species. In this case the use of the trinomial leads to confusion (as this is restricted to sub-species in the ICZN). I suggest that the denominations P. ovalecurtisi and P. ovalewallikeri should be used.

Line 113-115 References numbering is incorrect.

Figure 2. It is very difficult to distinguish between the various colours. Could I suggest that you restrict these to one colour for each geographic set: North Africa, West Africa, Central Africa, etc…. since there is no discussion as to variations between the parasites within each of these geographical groupings.

Line 249 Define IBD.

Line 481 Only 20 of the 22 genes are shown in bold in S2 Table.

Line 581-582 One cannot state that the cluster-specific adaptation signals are “dominated” by host-interaction genes (22 out of 315 is hardly a dominant proportion) The word to use should be “comprise”.

Line 584 There is no Figure 4C.

Line 617 It will be more correct to replace “challenge” by “add to”.

R11. We thank the reviewer for these minor comments, which have enabled us to improve the quality of the text and figures in the manuscript.
