# Supplementary material for: Identification of two genomic cryptotypes of Plasmodium malariae in Africa
Source: PLoS Negl Trop Dis. 2026 Jul 15;20(7):e0014176. doi: 10.1371/journal.pntd.0014176 (PMC13387610; doi:10.1371/journal.pntd.0014176)
Supplement: Peer review history

# Peer review: Decision Letter 0

**DOI:** 10.1371/journal.pntd.0014176.r001

**Authors:** Sanjai Kumar, Hira L Nakhasi

19 May 2026

PNTD-D-26-00567

Identification of two genomic cryptotypes of *Plasmodium malariae*  in Africa

PLOS Neglected Tropical Diseases

Dear Dr. Lefebvre,

Your manuscript has been seen by two experts in the field. After careful consideration, we feel that there are concerns that need to be fully addressed. Therefore, we invite you to submit a revised version of the manuscript that addresses the points raised during the review process.

Please submit your revised manuscript within by Jul 18 2026 11:59PM. If you will need more time than this to complete your revisions, please reply to this message or contact the journal office at plosntds@plos.org. When you're ready to submit your revision, log on to https://www.editorialmanager.com/pntd/ and select the 'Submissions Needing Revision' folder to locate your manuscript file.

Please include the following items when submitting your revised manuscript:

* A letter that responds to each point raised by the editor and reviewer(s). You should upload this letter as a separate file labeled 'Response to Reviewers'. This file does not need to include responses to any formatting updates and technical items listed in the 'Journal Requirements' section below.

* A marked-up copy of your manuscript that highlights changes made to the original version. You should upload this as a separate file labeled 'Revised Manuscript with Track Changes'.

* An unmarked version of your revised paper without tracked changes. You should upload this as a separate file labeled 'Manuscript'.

If you would like to make changes to your financial disclosure, competing interests statement, or data availability statement, please make these updates within the submission form at the time of resubmission. Guidelines for resubmitting your figure files are available below the reviewer comments at the end of this letter.

As the corresponding author, your ORCID iD is verified in the submission system and will appear in the published article. PLOS supports the use of ORCID, and we encourage all coauthors to register for an ORCID iD and use it as well. Please encourage your coauthors to verify their ORCID iD within the submission system before final acceptance, as unverified ORCID iDs will not appear in the published article. Only the individual author can complete the verification step; PLOS staff cannot verify ORCID iDs on behalf of authors.

We look forward to receiving your revised manuscript.

Kind regards,

Sanjai Kumar

Guest Editor

PLOS Neglected Tropical Diseases

Hira Nakhasi

Section Editor

PLOS Neglected Tropical Diseases

Shaden Kamhawi

co-Editor-in-Chief

PLOS Neglected Tropical Diseases

orcid.org/0000-0003-4304-636XX

Paul Brindley

co-Editor-in-Chief

PLOS Neglected Tropical Diseases

orcid.org/0000-0003-1765-0002

**Journal Requirements:**

1) We ask that a manuscript source file is provided at Revision. Please upload your manuscript file as a .doc, .docx, .rtf or .tex. If you are providing a .tex file, please upload it under the item type u2018LaTeX Source Fileu2019 and leave your .pdf version as the item type u2018Manuscriptu2019.

2) Please upload all main figures as separate Figure files in .tif or .eps format. For more information about how to convert and format your figure files please see our guidelines:

https://journals.plos.org/plosntds/s/figures

3) We have noticed that you have uploaded Supporting Information files, but you have not included a list of legends. Please add a full list of legends for your Supporting Information files after the references list.

4) Some material included in your submission may be copyrighted. According to PLOS's copyright policy, authors who use figures or other material (e.g., graphics, clipart, maps) from another author or copyright holder must demonstrate or obtain permission to publish this material under the Creative Commons Attribution 4.0 International (CC BY 4.0) License used by PLOS journals. Please closely review the details of PLOS's copyright requirements here: <a data-cke-saved-href="https://journals.plos.org/plosntds/s/licenses-and-copyright" href="https://journals.plos.org/plosntds/s/licenses-and-copyright">PLOS Licenses and Copyright</a>. If you need to request permissions from a copyright holder, you may use the&nbsp;<a data-cke-saved-href="https://storage.googleapis.com/plos-published-prod/c4ff/Content%20Copyright%20Permission%20Form.pdf?X-Goog-Algorithm=GOOG4-RSA-SHA256&X-Goog-Credential=wombat-sa%40plos-prod.iam.gserviceaccount.com%2F20220817%2Fauto%2Fstorage%2Fgoog4_request&X-Goog-Date=20220817T210608Z&X-Goog-Expires=86400&X-Goog-SignedHeaders=host&X-Goog-Signature=cd9dc868773378f7033b9f8cfb91430167d6275df90bce01342a454f36512530b64f24736fbf68c4fd96da74d663b6ee95b3420bded8fb0e833af2390b896b8706643b476d7f532408921b60576d65b60617070900fd2bcd28eec4cd32fefcf007d3655df17fcc91023329a28479ce0d1c5758174eedcc9e3c1788c5f7dd8523f3ddd871e02c998a20f7dc78ec00f313d4b5a78ba2037a6c7ef0a05dc29817da9368065460c8d8446659f35f0458fefabd3101eb7f926e9cb7a0b53e7bbe47aaed3e0ba52f9af9c2b8f6d7d3a8871b205fc4cbe97384ed37c7604fd7eecfedccf49cba12820b2e3b649541cefde3103cd7d72731b3e8b0248439b4cba691ca8a" href="https://storage.googleapis.com/plos-published-prod/c4ff/Content%20Copyright%20Permission%20Form.pdf?X-Goog-Algorithm=GOOG4-RSA-SHA256&X-Goog-Credential=wombat-sa%40plos-prod.iam.gserviceaccount.com%2F20220817%2Fauto%2Fstorage%2Fgoog4_request&X-Goog-Date=20220817T210608Z&X-Goog-Expires=86400&X-Goog-SignedHeaders=host&X-Goog-Signature=cd9dc868773378f7033b9f8cfb91430167d6275df90bce01342a454f36512530b64f24736fbf68c4fd96da74d663b6ee95b3420bded8fb0e833af2390b896b8706643b476d7f532408921b60576d65b60617070900fd2bcd28eec4cd32fefcf007d3655df17fcc91023329a28479ce0d1c5758174eedcc9e3c1788c5f7dd8523f3ddd871e02c998a20f7dc78ec00f313d4b5a78ba2037a6c7ef0a05dc29817da9368065460c8d8446659f35f0458fefabd3101eb7f926e9cb7a0b53e7bbe47aaed3e0ba52f9af9c2b8f6d7d3a8871b205fc4cbe97384ed37c7604fd7eecfedccf49cba12820b2e3b649541cefde3103cd7d72731b3e8b0248439b4cba691ca8a">PLOS Content Copyright Permission&nbsp;Form</a>

Please respond directly to this email and provide any known details concerning your material's license terms and permissions required for reuse, even if you have not yet obtained copyright permissions or are unsure of your material's copyright compatibility. Once you have responded and addressed all other outstanding technical requirements, you may resubmit your manuscript within Editorial Manager.

Potential Copyright Issues:

- Figures 1 and 3: Please (a) provide a direct link to the base layer of the map (i.e., the country or region border shape) and ensure this is also included in the figure legend; and (b) provide a link to the terms of use / license information for the base layer image or shapefile. We cannot publish proprietary or copyrighted maps (e.g. Google Maps, Mapquest) and the terms of use for your map base layer must be compatible with our CC BY 4.0 license.

Note: if you created the map in a software program like R or ArcGIS, please locate and indicate the source of the basemap shapefile onto which data has been plotted.

If your map was obtained from a copyrighted source please amend the figure so that the base map used is from an openly available source. Alternatively, please provide explicit written permission from the copyright holder granting you the right to publish the material under our CC BY 4.0 license.

If you are unsure whether you can use a map or not, please do reach out and we will be able to help you. The following websites are good examples of where you can source open access or public domain maps:

* U.S. Geological Survey (USGS) - All maps are in the public domain. (http://www.usgs.gov)

* PlaniGlobe - All maps are published under a Creative Commons license so please cite u201cPlaniGlobe, http://www.planiglobe.com, CC BY 2.0u201d in the image credit after the caption. (http://www.planiglobe.com/?lang=enl)

* Natural Earth - All maps are public domain. (http://www.naturalearthdata.com/about/terms-of-use/).

5) Please amend your detailed Financial Disclosure statement. This is published with the article. It must therefore be completed in full sentences and contain the exact wording you wish to be published.

State what role the funders took in the study. If the funders had no role in your study, please state: "The funders had no role in study design, data collection and analysis, decision to publish, or preparation of the manuscript.".

If you did not receive any funding for this study, please simply state: u201cThe authors received no specific funding for this work.u201d

**Reviewers' Comments:**

Reviewer's Responses to Questions

**Key Review Criteria Required for Acceptance?**

As you describe the new analyses required for acceptance, please consider the following:

**Methods**

-Are the objectives of the study clearly articulated with a clear testable hypothesis stated?

-Is the study design appropriate to address the stated objectives?

-Is the population clearly described and appropriate for the hypothesis being tested?

-Is the sample size sufficient to ensure adequate power to address the hypothesis being tested?

-Were correct statistical analysis used to support conclusions?

-Are there concerns about ethical or regulatory requirements being met?

Reviewer #1: The joining of the NHP and human data doesn’t make clear sense. There is a very solid analysis of P. malariae population structure in humans in Africa and a novel observation of P. brasilianum in NHPs in South America. Aside from the observation that there is P. brasilianum in NHPs there seems to be no real value from including the P. brasilianum data here. The major finding (and title) of the paper is not reliant on this data and it is confusing to include here. I would suggest the authors consider whether the article would be better served by separating these two datasets and only focusing on the malariae data here.

Reviewer #2: There are no concerns with respect to the methodology.

**********

**Results**

-Does the analysis presented match the analysis plan?

-Are the results clearly and completely presented?

-Are the figures (Tables, Images) of sufficient quality for clarity?

Reviewer #1: Analysis of the population structure defining cryptotypes

Definition – can the authors clarify what they mean by cryptotype in this context? The P. falciparum observation was a small number of loci around the genome where LD was maintained despite being on different chromosomes. In P. ovale the observation is that there are two “sibling” species which are sympatric but rarely recombine. The identification of segregation across the first few PCs seems closer to the ovale scenario, though latter data less so. A very clear definition of what is expected for each scenario would be valuable.

Driving factors – it is stated that no biological or geographical criteria allow these clusters to be distinguished. What factors were assessed, in addition what technical factors were assessed (i.e. sequencing depth, sequencing batch, instrument, etc). Do the clusters related to transmission intensity, mosquito species prevalence or historical use of antimalarials?

Figure 4: Was K=2 the best performing value of K?

Line 444-453: Did you examine if the selection of samples results in a geographical separation. There is higher yellow ancestry in East Africa than West Africa. Could the samples used for this analysis be marked on Fig. 3?

You state in the discussion that haplotype based signatures of selection are powerful for detecting recent events – why were they not used here?

Line 129: densify should be replaced with an alternative word.

Figure 3: If the best K is 6, why are the pie charts shown from K=3?

Figure 4: Using shades of red and yellow to define countries and red and yellow to define groups which are not related to geography is confusing. Could the country colors come from a different palate?

Reviewer #2: The analyses match the analytical plans, and the results clearly and fully presented, as are the figures. (minor suggestions are made below)

**********

**Conclusions**

-Are the conclusions supported by the data presented?

-Are the limitations of analysis clearly described?

-Do the authors discuss how these data can be helpful to advance our understanding of the topic under study?

-Is public health relevance addressed?

Reviewer #1: Conclusions and Discussion cover all these areas

Reviewer #2: The conclusions are supported by the data, and the limitations indicated. (See "Summary and General Comments")

**********

**Editorial and Data Presentation Modifications?**

Use this section for editorial suggestions as well as relatively minor modifications of existing data that would enhance clarity. If the only modifications needed are minor and/or editorial, you may wish to recommend “Minor Revision” or “Accept”.

Reviewer #1: (No Response)

Reviewer #2: See "Summary and General Comments"

**********

**Summary and General Comments**

Use this section to provide overall comments, discuss strengths/weaknesses of the study, novelty, significance, general execution and scholarship. You may also include additional comments for the author, including concerns about dual publication, research ethics, or publication ethics. If requesting major revision, please articulate the new experiments that are needed.

Reviewer #1: (No Response)

Reviewer #2: It is becoming increasingly clear that infections by P. malariae are by no means rare (at least in Africa). It is also known that these parasites cause infections that can persist for years. Thus, it is becoming important to study this species in the context of efforts to eliminate malaria. Yet, this parasite species has been relatively poorly studied, and molecular investigations focusing on this parasite are few.

The authors present a very interesting set of analyses of P. malariae populations, based on whole genome sequences, that substantially extends and improves on a recent analysis by Ibrahim et al. (2024). In addition to previous sequenced genomes, those of 45 new isolates from Africa were added, and of particular novelty the authors provide the genomes of two additional P. brasilianum isolates one from Colombia and another from French Guiana.

Their analyses confirmed previous observations concerning the differences between the populations of P. malariae across the continents and interestingly revealed the occurrence of two sympatric genetically distinct groups within the African isolates, which the authors consider as cryptotypes that nonetheless recombine.

The manuscript is well written, the data is clearly and convincingly presented, and fully supports the conclusions drawn by the authors. The salient critique I have is that the discussion presented in the manuscript deserves to be expanded to confront the data with some of the parasitological parameters of P. malariae. As it stands, the discussion generally reiterates the results presented, with little substantive speculation as to the nature or origin of the two cryptotypes.

General comments

- Why were the two P. malariae-like parasites (Rutledge et al. 2017), as well as the P. brasilianum genome that was previously obtained from a Bolivian strain (Bajic et al. 2022), not included with the other 179 isolates in the main analysis? These omissions are regrettable and somewhat weaken the manuscript, and I suggest that they should be added. This is because it would have helped resolve the phylogeny of the P. malariae-like parasite from chimpanzees and to provide a comparison of the 3 P. brasilianum isolates that were collected in distant geographical regions.

- The speculations concerning the cause of divergence between the two cryptotypes are based on the identifications of 315 genes that appear to have been differentially selected in the two populations (112 Y and 203 for R). For the discussion the authors selected only 22 of these genes, as they are known to be, or extrapolated as, implicated in host-parasite interactions. Speculation as to the nature of the selective forces were limited to a few lines (587-596). This is a major weakness of this manuscript, since this differential selection is what is the most interesting aspect of the manuscript. It is clear that the authors cannot speculate on the vast majority of the genes they identified since their function is unknown or only ascribed putatively. However, there are many avenues that would have been important to discuss.

First, there are numerous antigens that are considered to be important in the acquisition/evasion of immunity with some displaying significant diversity. It is of interest that none were identified in the analysis presented in S2 Table. It would have been of distinct interest to compare the diversity of such genes between the isolates from the two clusters (presumably these could be easily identified from the whole genome sequences).

Second, what the evidence that the two cryptotypes recombine. Is it possible that recombination is rare? Are mixed infections (Y+R) common? If the two types recombine frequently, one would expect them to merge into a single type eventually. If they do not, might this be a vase of speciation? This deserves speculation.

Third, there are some data on the transmission of P. malariae (and P. brasilianum) by different mosquito species. Could the authors speculate as to the differences in the anopheline species that occur between the different regions of the African continent, and whether this could have contributed to the generation of two cryptotypes (admixture of the two parasite types could have been recent, as large population movement between West and East Africa is likely to be recent).

Fourth, the genetic make-up of the human hosts might have been another contributory factor.

Of note the distribution of haemoglobinopathies differ across the African continent. This is also the case for Duffy negativity. Indeed, there was recent speculation (Culleton et al. 2023 Trends in Parasitology, in which one of the co-authors of this manuscript is also a co-author) that P. malariae might preferentially invade reticulocytes. Although infection of Duffy negative individuals by P. malariae occurs, the course of the infection might differ between Duffy negative and Duffy positive persons. It would be of interest for the authors to indicate the ethnic origin of the patients from whom the samples were collected. Since nearly all the African samples collected were from persons returning to the UK or France, it is possible that some could have been of African descent while others not (I am aware that this might be difficult for the samples collected in the UK).

Minor comments

Line 89 It is now recognised that the population of P. ovale is actually composed of two distinct species. In this case the use of the trinomial leads to confusion (as this is restricted to sub-species in the ICZN). I suggest that the denominations P. ovalecurtisi and P. ovalewallikeri should be used.

Line 113-115 References numbering is incorrect.

Figure 2. It is very difficult to distinguish between the various colours. Could I suggest that you restrict these to one colour for each geographic set: North Africa, West Africa, Central Africa, etc…. since there is no discussion as to variations between the parasites within each of these geographical groupings.

Line 249 Define IBD.

Line 481 Only 20 of the 22 genes are shown in bold in S2 Table.

Line 581-582 One cannot state that the cluster-specific adaptation signals are “dominated” by host-interaction genes (22 out of 315 is hardly a dominant proportion) The word to use should be “comprise”.

Line 584 There is no Figure 4C.

Line 617 It will be more correct to replace “challenge” by “add to”.

**********

PLOS authors have the option to publish the peer review history of their article (what does this mean?). If published, this will include your full peer review and any attached files.

If you choose “no”, your identity will remain anonymous but your review may still be made public.

**Do you want your identity to be public for this peer review?**  For information about this choice, including consent withdrawal, please see our Privacy Policy.

Reviewer #1: No

Reviewer #2: No

[NOTE: If reviewer comments were submitted as an attachment file, they will be attached to this email and accessible via the submission site. Please log into your account, locate the manuscript record, and check for the action link "View Attachments". If this link does not appear, there are no attachment files.]

**Figure resubmission:**

While revising your submission, we strongly recommend that you use PLOS’s NAAS tool (https://ngplosjournals.pagemajik.ai/artanalysis) to test your figure files. NAAS can convert your figure files to the TIFF file type and meet basic requirements (such as print size, resolution), or provide you with a report on issues that do not meet our requirements and that NAAS cannot fix.

After uploading your figures to PLOS’s NAAS tool - https://ngplosjournals.pagemajik.ai/artanalysis, NAAS will process the files provided and display the results in the "Uploaded Files" section of the page as the processing is complete. If the uploaded figures meet our requirements (or NAAS is able to fix the files to meet our requirements), the figure will be marked as "fixed" above. If NAAS is unable to fix the files, a red "failed" label will appear above. When NAAS has confirmed that the figure files meet our requirements, please download the file via the download option, and include these NAAS processed figure files when submitting your revised manuscript.

**Reproducibility:**

To enhance the reproducibility of your results, we recommend that authors of applicable studies deposit laboratory protocols in protocols.io, where a protocol can be assigned its own identifier (DOI) such that it can be cited independently in the future. Additionally, PLOS ONE offers an option to publish peer-reviewed clinical study protocols. Read more information on sharing protocols at https://plos.org/protocols?utm_medium=editorial-email&utm_source=authorletters&utm_campaign=protocols

# Author comment: Author response to Decision Letter 1

**DOI:** 10.1371/journal.pntd.0014176.r002

15 Jun 2026

Attachment Submitted filename: Answer_reviews.pdf

# Peer review: Decision Letter 1

**DOI:** 10.1371/journal.pntd.0014176.r003

**Authors:** Sanjai Kumar, Hira L Nakhasi

2 Jul 2026

Dear Dr. Lefebvre,

We are pleased to inform you that your manuscript 'Identification of two genomic cryptotypes of *Plasmodium malariae*  in Africa' has been provisionally accepted for publication in PLOS Neglected Tropical Diseases.

Before your manuscript can be formally accepted you will need to complete some formatting changes, which you will receive in a follow up email. A member of our team will be in touch with a set of requests.

Please note that your manuscript will not be scheduled for publication until you have made the required changes, so a swift response is appreciated.

IMPORTANT: The editorial review process is now complete. PLOS will only permit corrections to spelling, formatting or significant scientific errors from this point onwards. Requests for major changes, or any which affect the scientific understanding of your work, will cause delays to the publication date of your manuscript.

Should you, your institution's press office or the journal office choose to press release your paper, you will automatically be opted out of early publication. We ask that you notify us now if you or your institution is planning to press release the article. All press must be co-ordinated with PLOS.

Thank you again for supporting Open Access publishing; we are looking forward to publishing your work in PLOS Neglected Tropical Diseases.

Best regards,

Sanjai Kumar

Guest Editor

PLOS Neglected Tropical Diseases

Hira Nakhasi

Section Editor

PLOS Neglected Tropical Diseases

Shaden Kamhawi

co-Editor-in-Chief

PLOS Neglected Tropical Diseases

orcid.org/0000-0003-4304-636XX

Paul Brindley

co-Editor-in-Chief

PLOS Neglected Tropical Diseases

orcid.org/0000-0003-1765-0002

***********************************************************

p.p1 {margin: 0.0px 0.0px 0.0px 0.0px; line-height: 16.0px; font: 14.0px Arial; color: #323333; -webkit-text-stroke: #323333}span.s1 {font-kerning: none

Reviewer's Responses to Questions

**Key Review Criteria Required for Acceptance?**

As you describe the new analyses required for acceptance, please consider the following:

**Methods**

-Are the objectives of the study clearly articulated with a clear testable hypothesis stated?

-Is the study design appropriate to address the stated objectives?

-Is the population clearly described and appropriate for the hypothesis being tested?

-Is the sample size sufficient to ensure adequate power to address the hypothesis being tested?

-Were correct statistical analysis used to support conclusions?

-Are there concerns about ethical or regulatory requirements being met?

Reviewer #1: (No Response)

Reviewer #2: The authors adequately addressed all the comments

**********

**Results**

-Does the analysis presented match the analysis plan?

-Are the results clearly and completely presented?

-Are the figures (Tables, Images) of sufficient quality for clarity?

Reviewer #1: (No Response)

Reviewer #2: The authors adequately addressed all the comments

**********

**Conclusions**

-Are the conclusions supported by the data presented?

-Are the limitations of analysis clearly described?

-Do the authors discuss how these data can be helpful to advance our understanding of the topic under study?

-Is public health relevance addressed?

Reviewer #1: (No Response)

Reviewer #2: The authors adequately addressed all the comments

**********

**Editorial and Data Presentation Modifications?**

Use this section for editorial suggestions as well as relatively minor modifications of existing data that would enhance clarity. If the only modifications needed are minor and/or editorial, you may wish to recommend “Minor Revision” or “Accept”.

Reviewer #1: (No Response)

Reviewer #2: (No Response)

**********

**Summary and General Comments**

Use this section to provide overall comments, discuss strengths/weaknesses of the study, novelty, significance, general execution and scholarship. You may also include additional comments for the author, including concerns about dual publication, research ethics, or publication ethics. If requesting major revision, please articulate the new experiments that are needed.

Reviewer #1: The authors have appropriately addressed all comments in the revised manuscript

Reviewer #2: The authors adequately addressed all the comments. The manuscript is suitable for publication

**********

PLOS authors have the option to publish the peer review history of their article (what does this mean?). If published, this will include your full peer review and any attached files.

If you choose “no”, your identity will remain anonymous but your review may still be made public.

**Do you want your identity to be public for this peer review?**  For information about this choice, including consent withdrawal, please see our Privacy Policy.

Reviewer #1: No

Reviewer #2: No

# Editor report: Acceptance letter

**DOI:** 10.1371/journal.pntd.0014176.r004

**Authors:** Sanjai Kumar, Hira L Nakhasi

Dear Ms Lefebvre,

We are delighted to inform you that your manuscript, "Identification of two genomic cryptotypes of *Plasmodium malariae*  in Africa," has been formally accepted for publication in PLOS Neglected Tropical Diseases.

We have now passed your article onto the PLOS Production Department who will complete the rest of the publication process. All authors will receive a confirmation email upon publication.

The corresponding author will soon be receiving a typeset proof for review, to ensure errors have not been introduced during production. Please review the PDF proof of your manuscript carefully, as this is the last chance to correct any scientific or type-setting errors. Please note that major changes, or those which affect the scientific understanding of the work, will likely cause delays to the publication date of your manuscript. Note: Proofs for Front Matter articles (Editorial, Viewpoint, Symposium, Review, etc...) are generated on a different schedule and may not be made available as quickly.

Soon after your final files are uploaded, the early version of your manuscript will be published online unless you opted out of this process. The date of the early version will be your article's publication date. The final article will be published to the same URL, and all versions of the paper will be accessible to readers.

For Research Articles, you will receive an invoice from PLOS for your publication fee after your manuscript has reached the completed accept phase. If you receive an email requesting payment before acceptance or for any other service, this may be a phishing scheme. Learn how to identify phishing emails and protect your accounts at https://explore.plos.org/phishing.

Thank you again for supporting open-access publishing; we are looking forward to publishing your work in PLOS Neglected Tropical Diseases.

Best regards,

Shaden Kamhawi

co-Editor-in-Chief

PLOS Neglected Tropical Diseases

Paul Brindley

co-Editor-in-Chief

PLOS Neglected Tropical Diseases
